# Models of spatial analysis for vector-borne diseases studies: A systematic review

**DOI:** 10.14202/vetworld.2022.1975-1989

**Published:** 2022-08-19

**Authors:** Licet Paola Molina-Guzmán, Lina A. Gutiérrez-Builes, Leonardo A. Ríos-Osorio

**Affiliations:** 1 Grupo Biología de Sistemas, Escuela de Ciencias de la Salud, Facultad de Medicina, Universidad Pontificia Bolivariana, Medellín, Colombia; 2 Grupo de Investigación Salud y Sostenibilidad, Escuela de Microbiología, Universidad de Antioquia UdeA, Calle 70 No. 52-21, Medellin - Colombia

**Keywords:** disease vectors, geographic information systems, medical, spatial analysis, topography

## Abstract

**Background and Aim:**

Vector-borne diseases (VBDs) constitute a global problem for humans and animals. Knowledge related to the spatial distribution of various species of vectors and their relationship with the environment where they develop is essential to understand the current risk of VBDs and for planning surveillance and control strategies in the face of future threats. This study aimed to identify models, variables, and factors that may influence the emergence and resurgence of VBDs and how these factors can affect spatial local and global distribution patterns.

**Materials and Methods:**

A systematic review was designed based on identification, screening, selection, and inclusion described in the research protocols according to the preferred reporting items for systematic reviews and meta-analyses guide. A literature search was performed in PubMed, ScienceDirect, Scopus, and SciELO using the following search strategy: Article type Original research, Language: English, Publishing period: 2010–2020, Search terms: Spatial analysis, spatial models, VBDs, climate, ecologic, life cycle, climate variability, vector-borne, vector, zoonoses, species distribution model, and niche model used in different combinations with “AND” and “OR.”

**Results:**

The complexity of the interactions between climate, biotic/abiotic variables, and non-climate factors vary considerably depending on the type of disease and the particular location. VBDs are among the most studied types of illnesses related to climate and environmental aspects due to their high disease burden, extended presence in tropical and subtropical areas, and high susceptibility to climate and environment variations.

**Conclusion:**

It is difficult to generalize our knowledge of VBDs from a geospatial point of view, mainly because every case is inherently independent in variable selection, geographic coverage, and temporal extension. It can be inferred from predictions that as global temperatures increase, so will the potential trend toward extreme events. Consequently, it will become a public health priority to determine the role of climate and environmental variations in the incidence of infectious diseases. Our analysis of the information, as conducted in this work, extends the review beyond individual cases to generate a series of relevant observations applicable to different models.

## Introduction

Vector-borne diseases (VBDs) constitute a complex global health problem for humans and animals [1, 2]. Multiple infectious agents, hosts, and vectors are involved in these diseases and their epidemiology [3]. Disease patterns, which vary in different geographic zones, may change over time and space. Multiple environmental, ecologic, social, economic, and political factors may foster the interactions between infectious agents, their many hosts and vectors, and human beings [4, 5].

Emerging pathogens described in various regions have been imported to areas where they were not previously found. The term VBD is also used to describe agents that were consistently present within an affected area at a low level or infected a different host and, due to a change, now extend to a broader area within a population or organisms not previously recognized as hosts [6, 7]. Information describing the spatial distribution of species of different vectors and their relationship with environmental conditions where they grow is critical to understanding the current risk of transmissible diseases and planning surveillance and control strategies in the face of future threats [8–10].

In addition to microorganisms responsible for VBDs, a series of risk factors that create a predisposition, to a smaller or greater extent, to disease emergence or expansion can also be identified [11]. Global warming and environmental changes (modification of abiotic elements, i.e., the climate, and biotic elements, i.e., animals and plants) are among the ecosystem factors that may favor VBD emergence and resurgence. While the relationship between climate change and the emergence of infectious diseases has been widely discussed, ecosystem changes should also be addressed because climate and environmental changes and their interrelation impact ecosystem transformations [12]. The troubling role that climate and landscapes play in VBDs should be highlighted due to the apparent increasing pressure they exert on the distribution of vectors and microorganisms. Changes to these factors depend significantly on human activity and the natural environment. Various techniques selected according to the nature of proposed hypotheses and their predictive capacity is used to model the spatial distribution of species [13]. The general objective of species distribution models is to identify the relationship between a particular species (absence/presence) and environmental data (e.g., meteorological data, coverage of soil use, and remote sensing data) and use the knowledge obtained from the areas examined to make predictions for non-sampled locations in an analogous region [14, 15].

This study aimed to identify models, variables, and factors that may influence the emergence and resurgence of VBDs and how these factors can affect spatial local and global distribution patterns.

## Materials and Methods

### Ethical approval

This study was approved by the Bioethics Committee of the Universidad Pontificia Bolivariana.

### Study period

The study was conducted on February 13, 2021, and it was designed to cover scientific literature published during the period 2010–2020.

### Search strategy

In 2021, a systematic literature search was performed in the ScienceDirect, Scopus, PubMed, and SciELO databases using the Preferred Reporting Items for Systematic Reviews and Meta-Analyses methodology [16] and the systematic review methodology proposed by Cardona-Arias *et al*. [17]. To grant exhaustivity for the search protocol, a query was performed using non-*Descriptores de Ciencias de la Salud* (DeCS) descriptors (Health Sciences Descriptors [DeCS]), by sensibility using thesaurus terms, DeCS descriptors or Medical Subject Headings, and by specificity utilizing a combination with Boolean operators of the search terms defined according to the research question: “What are the models, variables, and temporary risk factors intervening in the proliferation of VBDs for their spatial analysis?”

A search was performed with a general path (“spatial analysis” OR “spatial models”), using the operators “AND ALL” or “AND” with the search terms (“vector-borne diseases” OR “climate” OR “ecologic” OR “life cycle” OR “climate variability” OR “vector-borne” OR “vector” OR “zoonoses” OR “species distribution model” OR “niche model”). For ScienceDirect, Scopus, PubMed, and SciELO databases, we used the time limits “2010 to present,” “published 2010 to present,” “published in the last 10 years,” and “últimos ten años,” to cover scientific literature published during the period 2010–2020. The specific search paths used in the four databases were the following:

#### ScienceDirect

TITLE-ABSTR-KEY (“spatial analysis” OR “spatial models”), and ALL (“vector-borne diseases” OR “climate” OR “ecologic” OR “life cycle” OR “climate variability” OR “vector-borne” OR “vector” OR “zoonoses” OR “species distribution model” OR “niche model”).

#### PubMed

(“Spatial analysis” [Title/Abstract] OR “spatial models” [Title/Abstract]) AND (“vector-borne diseases” OR “climate” OR “ecologic” OR “life cycle” OR “climate variability” OR “vector-borne” OR “vector” OR “zoonoses” OR “species distribution model” OR “niche model”).

#### Scopus

TITLE-ABS-KEY (“spatial analysis” OR “spatial models”) AND ALL (“vector-borne diseases” OR “climate” OR “ecologic” OR “life cycle” OR “climate variability” OR “vector-borne” OR “vector” OR “zoonoses” OR “species distribution model” OR “niche model”).

### SciELO

“Spatial analysis” OR “spatial models” [Abstract] and “vector-borne diseases” OR “climate” OR “ecologic” OR “life cycle” OR “climate variability” OR “vector-borne” OR “vector” OR “zoonoses” OR “species distribution model” OR “niche model” [All indexes].

The citations found with their respective abstracts were imported to reference management software (Mendeley Ltd., version 2017, New York, USA) which helped eliminate duplicate references among databases.

### Inclusion and exclusion criteria

**Figure-1:**
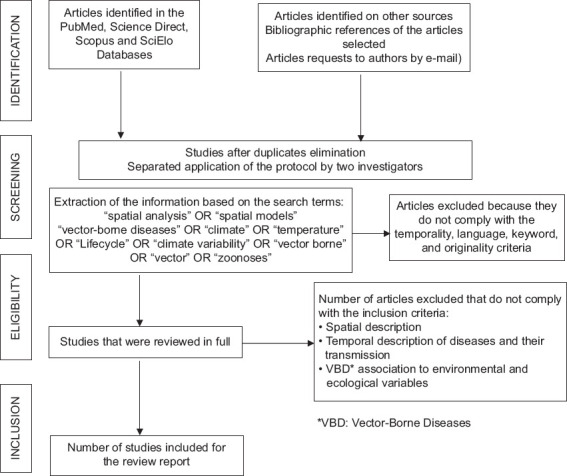
Methodologic design for the systematic review.

Only original scientific articles published in the past 10 years (2010–2020) written in English that used a methodology for the analysis of the spatial and temporal distribution of disease vectors and disease transmission and studies evaluating VBDs in association with environmental variables were considered. We excluded articles with methodologies using *in vitro* models or cell cultures as units of analysis, studies based only on laboratory tests, and studies using models based only on the incidence of diseases or risk maps without vector information. Application of the research protocol (Figure-1) and verification of inclusion and exclusion criteria were performed independently by two investigators using an Excel form to ensure review reproducibility. Discrepancies were resolved by consensus.

Data from each publication were extracted and tabulated for subsequent analysis using a collection form that contained general information about the articles (Title, Journal, Publishing Year, and Country of Origin of the data). The reports were described using the following categories: Vector species and primary associated disease, study area, biological data type, environmental data type, the method applied for the Geographic Information System, projection types analyzed in greater detail regarding biological (number of records, data source), and environmental (number of variables and approximate spatial resolution) data, methods (algorithm used, and models based on different algorithms), and temporal projections (years, general circulation model, and climate change scenario).

### Thematic network analysis

A descriptive methodology was used for thematic analysis based on the network approach [18, 19]. The information was obtained from the scientific literature, and themes and connections between two or more variables were identified. For this identification, a link matrix was created. Relationship evidence was compiled and classified according to the intensity and the interaction frequency. The number of links identified between variables and their closeness based on the preliminary classification information obtained from the literature was evaluated. An investigation was conducted in specialized databases, searching for file records and link experiences between at least two identified variables. The level of thematic density, analytic input, intermediation, modularity, and closeness of each variable was determined in relation to other variables and the network itself, based on the information entered through a matrix. A network graphic representation was developed, showing networks of variables related to a single theme or an environment using free software (Gephi^©^ Version 0.9.2 2017–2018, www.Gephi.org).

## Results and Discussion

### Literature search

**Figure-2:**
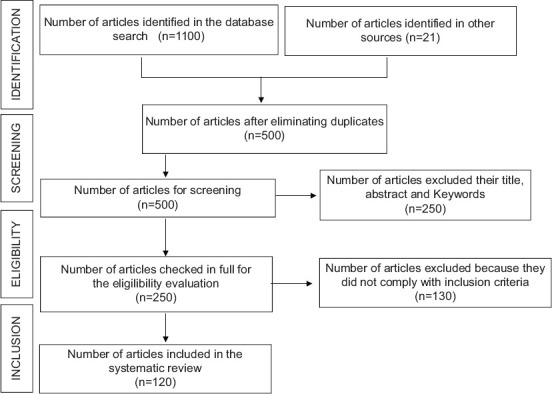
Algorithm used for article selection.

The search protocol used in the databases described resulted in the identification of 1100 articles published between 2010 and 2020, plus 21 articles identified in the bibliographic references of those articles. Subsequently, 621 duplicate references across the databases were eliminated with the help of Mendeley® software. Eventually, 500 publications were evaluated according to their title, abstract, and keywords, of which 250 were discarded because they did not comply with the inclusion criteria defined according to the research question. The full texts of 250 articles were analyzed, and 130 were eliminated because they met the exclusion criteria. Ultimately, the implementation of the search protocol in the four databases led to the systematic review of 120 articles (Figure-2).

### Distribution of scientific articles according to their year of publication

For many decades, the importance and distribution of VBDs have been widely recognized [20], as evidenced by the constant publishing of VBD-related research involving distribution and spatiality over the past 10 years.

**Figure-3:**
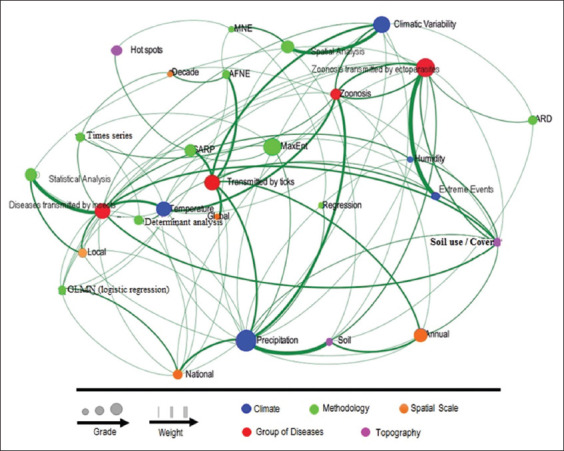
Thematic network. Each node represents a thematic term, and its size indicates the level of connectivity. The thickness of curved lines constitutes a connector that shows the frequency of this relationship. MNE=Ecological niche modeling, ENFA=Ecological niche factorial analysis, ARD=Distance-based regression tree, GARP=Genetic algorithm for rule set production.

The network of connections between the types of diseases, climate variables, soil features, methodologies, and spatial and temporal scales is shown in Figure-3. Among the most studied topics, the thematic network highlights temperature, climate variability, precipitation, and diseases transmitted by various vectors, particularly those transmitted annually by insects on a local scale. Time series and regressions are among the least used statistical tools for data analysis, providing weak connections with thematic terms from other categories of statistical analysis; thus, a trend toward reducing the use of these approaches was observed.

In general, the relative simplicity of statistical methods constitutes an advantage. However, their applicability in modeling infectious disease processes is linear, making it challenging to identify interactions between a pathogenic microorganism, its climate setting, and the host [21]. On the contrary, models based on the spatial and temporal distribution (discriminant analysis, the generalized linear model (GLM), and the random forest method) can capture the dynamic nature of these processes and transmission patterns in the most realistic way possible [22]. However, their complexity requires a thorough knowledge of the specific characteristics of the disease [23]. The significant number of climate parameters and behavioral response variables is also challenging for the construction and understanding of mechanistic models, as these work under the assumption that a complex system can be understood by examining the function of each of its parts and the combination between them, which usually have a physical, tangible appearance, with accurate, solid, and visible components [24].

Nevertheless, many types of infectious disease models have been developed based on mechanistic approaches, such as subpopulation modeling [25], the probabilistic spatial-temporal model [21], spatial phylogenetic analyses [26], and vectorial capacity models [27]. In this systematic review, we observed that their application in VBDs is still limited, particularly on a continental and global scale, which could be due to the insufficient number of large-scale studies involving mechanistic models. This is not the case for diseases whose natural transmission history and epidemiology have been investigated for a long time, for example, malaria, as they can be studied using these models because of the availability of essential data.

While VBDs transmitted by insects were predominantly discussed, studies on diseases transmitted by ticks have increased in recent years [28]. Research trends show a variety of patterns in each variable relationship category (Figure-3). Regarding topographic and climate variables related to the distribution of VBDs, when analyzed using the data extracted from publications on each disease and vector involved and comparing their response to such variables, we observed that in the last decade, topographic and climate factors have influenced the distribution of VBDs. The emergence of VBDs is positively or negatively related to these factors at different spatial scales (local, national, global, and cross-border) and with different vectors (Table-1).

**Table-1:** Summary of the number of studies, classified in four distinct spatial scales regarding climatic change and topographic variables and divided according to the type of response from vector-borne diseases.

Variable	Local	National	Cross-border	Global	%VBD^a^
Climatic Variability	15+; 6-; 6I	39+; 10-; 9I	8+; 3-; 6U	0	45% Mosquitoes 35% Ectoparasites^¥^ 28% Ticks
Extreme Events	15+; 2I	10+; 4-	2+	0	12% Mosquitoes 8% Ectoparasites
Humidity	24+; 12-; 8I	19+; 3-; 3I	0	1-; 1I	32% Ticks 25% Mosquitoes
Precipitation	48+; 11-; 11I	27+; 1-; 5I	6+; 2-; 2I	2I	18% Mosquitoes 7% Ectoparasites
Temperature	66+; 21-; 21I	61+; 9-; 18I	20+; 2-; 4I	8+; 2-	48% Mosquitoes 25% Ectoparasites 10% Ticks
Soil	12+;4I	5+;10+;5I	0	0	9% Mosquitoes 6% Ectoparasites
Soil Use/Cover	10+;4-	12+;5-;12I	0	2+	25% Ectoparasites 10% Mosquitoes 8% Ticks

The signs after a figure mean a positive effect (+), a negative effect (-), and an uncertain effect (I).

a Some studies evaluated more than one disease and its respective vector. Therefore, the total number of final studies included in the qualitative synthesis was 112.

¥ This refers to studies including vectors such as fleas, lice, triatomines, and flies. VBD=Vector-borne diseases

In addition, we found that zoonoses transmitted by ectoparasites (ticks, lice, fleas, and flies) show an increasing trend, both in positive and uncertain responses to changes in climate variables. As for diseases transmitted by insects, the number of articles reporting positive effects, negative responses, and uncertain effects on VBDs has increased, with evidence of the positive impact of climate variables on the distribution of infectious diseases increasing at a faster pace.

### Spatial models and variables that influence VBD distribution

In the studies analyzed, more than ten different modeling methods were used to predict vector distribution. The most used method was the Algorithm For Maximum Entropy (MaxEnt) [29], followed by GLM [30], genetic algorithms [31], Discriminant Analysis [32], Hot Spots [33], and CLIMEX. Other methods applied with less frequency were the Ecological Niche Factor Analysis, Boosted Regression Trees [34], BioClim [35], and Random Forests [36]. The articles analyzed were classified according to the following categories: vector species and primary associated disease, study area, biological and environmental data typology, and spatial method. The articles were analyzed in greater detail, considering biological data (number of records and data source), environmental data (number of variables and approximate special resolution), methods (algorithm and set models based on different algorithms), and future projections (years, general circulation model, and climate change scenario) (Table-2) [37–56].

**Table-2:** Leading models of spatial analysis for vector - borne diseases.

Models	Transmission	Type of Disease	Climatic variable	Spatial Scale	Time Scale	Location	Reference
Ecological Niche Modeling							
Maximum Entropy GARP* Hot Spots CLIMEX GLMM	Ectoparasites Transmitted by insects	African Trypanosomiasis; Diseases transmitted by ticks; Chagas Disease; Japanese Encephalitis; Arbovirus	Climate variability Temperature	National Cross - border	Decades	Senegal Canada USA Mexico Korea Australia	[37–41]
Mechanistic models							
Population model Vectorial Capacity model	Transmitted by insects Ectoparasites	Dengue fever; Malaria; Chagas Disease; Leishmaniasis	Temperature Precipitation	Local National	Decades Intra - annually	Colombia China Germany	[42,43]
Other statistical analysis							
Discriminant analysis; Likelihood ratio test	Transmitted by insects; Ectoparasites	Malaria; Lymphatic filariasis, Dengue fever, Arbovirus; Chagas Disease, Leishmaniasis	Climate variability	National Local	Decades Inter - annually	Netherlands Center and South America France	[44–46]
Spatial Analysis							
Hot Spots, Geospatial distribution analysis	Ectoparasites; Transmitted by insects; Transmitted by ticks	Chagas Disease; Leishmaniasis; Malaria, Dengue fever; Arbovirus; Lyme Disease	Temperature Climate variability	Local National	Intra - annually	Europe Kenya France	[47–49]
Time series analysis							
Generalized Linear Models	Transmitted by insects; Transmitted by ticks	Malaria; Dengue fever; West Nile fever; Rickettsia; Lyme Disease	Climate variability Temperature	Local National	Decades Inter - annually	New Zealand, China Bangladesh Tanzania Serbia	[50–53]
Regression							
Linear and Non - linear regression	Transmitted by insects; Transmitted by ticks	Malaria; Arbovirus; Dengue fever; Lyme Disease	Temperature Precipitation	Local	Decades Inter - annually	Southeast Asia China Italy	[54–56]

* GARP=Genetic algorithm for rule set production

The maximum entropy method, which is usually included in free software with a user-friendly interface and many parametrization options [34, 42], was the most used method to evaluate spatial distribution compared to the other algorithms used in many studies [57–59]. GLMs were the second most common spatial analysis method used. These methods offer more flexibility than automatic algorithms (e.g., MaxEnt and genetic algorithm for rule set production [GARP]), improving model adjustment and ecological interpretation of parameters [60]. The Hot Spots model has been used in ecological studies to help detect areas with a higher concentration and reiteration of vectors or diseases, as well as risk factors [61]. CLIMEX has already been described as a model based on the biological processes of vectors, such as life cycle period, biting rates, dispersion capacity, and temperature limits for larvae development. Including these data improve the prediction of the biological plausibility of models, but they require an adequate understanding of vector physiology, making parametrization more challenging [38, 62, 63]. Models produced through different algorithms (variables) may generate dissimilar and even contradicting results [64–67]. Pinto *et al*. [68] examined the effects of climate on dengue fever outbreaks in Singapore City using a multivariate Poisson regression model. They found that temperature explained a significant proportion of dengue outbreaks in this city. Conversely, Liu-Helmersson *et al*. [27] performed an analysis based on an autoregressive integrated moving average model using the same data source and did not find any significant association between temperature and dengue emergence [69].

Even though many models have been considered, independent evaluations could not identify a single algorithm that might be recommended for every analysis circumstance involving VBD spatialization [70, 71]. A valid alternative to avoid selecting a particular method is to test models generated by a set of algorithms [72] and pool results as a set model [73, 74]. Using a set of models generated by a series of algorithms, uncertainties can be adequately quantified, improving the study results [75, 76].

More than 70% of the publications (Tables-1 and 2) used a combination of different parameters (ecologic, climate, soil variables, disease type, and vector type). In some studies, it has been suggested that these variables are necessary to obtain comparable results. Khatchikian *et al*. [77] evaluated five models to identify high-density areas for *Aedes* *aegypti* in Bermuda. To map the potential spatial distribution, they used the BIOCLIM and DOMAIN models, analyzing variables such as climate factors (temperature, precipitation, and humidity) and records on the presence of the species. Furthermore, to determine the frequency of an event by repeated occurrence, they used the GARP, GLM (logistic regression), and MaxEnt models. Arboleda *et al*. [42] conducted a study to determine the distribution patterns of *A. aegypti* natural breeding sites in Colombia, analyzing climate-related variables (annual and monthly precipitation, topography, and altitude). In this study, the combination of different variables improved the detection of this mosquito species’ natural breeding sites, which helped optimize financial efforts and investment in programs for dengue fever control within the region.

On the other hand, Huntingford *et al*. [78] used the primary reproduction number method (R_0_) to identify VBD transmission and found it to be strongly determined by the extrinsic vector incubation period, which is extremely sensitive to temperature changes. However, to define incubation periods, they used the Hartemink *et al*. [44] method to calculate the percentage change in standard deviation for monthly temperature anomalies before and after 1980 for each geographical network, without considering exhaustively if the equation structure and parameters reflected the reality of the location and its scale [44]. Likewise, Mordecai *et al*. [79], in their Zika, dengue, and Chikungunya study conducted in Africa, found that temperature fluctuations may substantially alter a parasite’s incubation period. Thus, nowadays, it is considered helpful to consider a combination of different variables to bridge current research gaps and support effective designs to map disease transmission mechanisms.

### VBD geospatial projections and variables associated with vector distribution

Four vectors were identified in the 112 articles reviewed: Insects (59%), sandflies (28%), ticks (13%), and Tsetse flies (3%). The geographical scope of the studies examined varied from local to global. Studies considering the future projections of VBDs generally predict expansions as a response under scenarios of climate change, along with changes in external environmental conditions (Table-1). This trend can be observed across different taxonomic groups, as shown by long-term field studies, which have demonstrated that some species have recently moved to higher latitudes and altitudes as a response to global warming [80–85]. Regarding the methodology used, the distribution range of a species was a conditioning factor.

### Dengue: *A. aegypti* and *Aedes albopictus* (Diptera: *Culicidae*)

Currently distributed across most of the tropical regions of the world, *A. aegypti* is recognized as the main vector for dengue fever, with *A. albopictus* as the second most important vector [86]. In the articles, we evaluated regarding global distribution projections for 2030 and 2070; we found that most of the areas where the vector is currently present will maintain favorable climate conditions for its development. At the same time, new areas will also become suitable for expanding the vector distribution range, such as the inland region of Australia, the Arabian Peninsula, Southern Iran, and some areas of North America [87].

Other short-term future projections indicate that macroclimate conditions suitable for this vector’s development should start to expand between 2010 and 2039 [88]. In Brazil, models predict a reduction or contraction of this vector’s distribution range in the northern and northeastern regions, along with a potential expansion toward the south by 2050 [89]. Locally, as *A. aegypti* is a highly adaptable vector in urban settings;, its distribution may be influenced by the availability of artificial breeding sites, such as water tanks and swimming pools [90].

In Australia, studies based on models that only considered the analysis of climate variables did not detect any emergent site locations or dengue cases [91]. In Mexico, Moreno-Madriñán *et al*. [90], using remote sensing to detect the abundance of *A. aegypti* pupae, reported a significant positive correlation between the estimations of daytime and nighttime temperatures at different altitudes, and additionally a significant association between the estimated precipitation and the nighttime land surface temperature, more than the daytime temperature. They also observed that altitude plays an essential role in the abundance of pupae populations. In this study, the highest pupae numbers were found from 18 to 2000 meters above sea level (MASL), which is consistent with Quintero *et al*. [86], who reported the presence of this vector in areas at an altitude higher than 2200 MASL at Cáqueza, Cundinamarca, in Colombia.

### Malaria: *Anopheles* spp. (Diptera: *Culicidae*)

Malaria constitutes a public health problem worldwide. Female mosquitoes belonging to the genus *Anopheles* are the primary vector, and many species have been identified for transmission [61]. According to the global distribution described in the articles we evaluated, these vectors will expand toward the south and southeastern regions of the world, based on global warming scenarios [92–95]. When assessing mosquito survival rates through ecological niche models considering variables such as climate variability, breeding site availability, and life cycle development, Tonnang *et al*. [92] reported that countries in eastern Africa would maintain favorable climate conditions for these vectors in the coming decades, compared to countries in western Africa where conditions are not going to show extreme variations.

### Leishmaniasis: *Lutzomyia* spp. and *Phlebotomus* spp. (Diptera: Psychodidae)

Leishmaniasis is a tropical disease widely distributed in 98 countries, with approximately 0.2–0.4 million cases of visceral leishmaniasis and 0.7–1.2 million cases of cutaneous leishmaniasis reported annually [96]. According to their distribution, leishmaniasis vectors are classified into two genres: *Lutzomyia* in the Americas and *Phlebotomus* in other continents [96]. Regarding future projections and global distribution, vectors such as *Lutzomyia whitmani*, *Lutzomyia intermedia*, and *Lutzomyia migonei* could expand toward South America by 2050 [96] with expansion areas in different regions across the continent, but the most probable direction is southwards. On the contrary, in Colombia, future projections of regional distribution models suggest a reduction of the area of emergence for *Lutzomyia longipalpis* and *Lutzomyia evansi* that has been associated with changes in their altitude distribution, even when this vector can be present across a wide range of latitudes, from Mexico to Argentina [97]. Moo-Llanes *et al*. [98] reported an expansion toward the north, and 27 of the 28 species involved are currently present in Mexico, Guatemala, Belize, the United States, and Canada.

### Ticks as vectors (Acari: Ixodida)

Ticks are an important ectoparasite type that can infect domestic and wild animals and impact public health. A diverse group with approximately 898 recognized species, ticks are divided into three main families: *Argasidae* (194 species), *Ixodidae* (703 species), and Nuttalliellidae (1 species) [99–101]. These arthropods can infect a wide range of animals, including humans, who are accidental hosts of many species of ticks, such as *Amblyomma americanum*, *Ixodes ricinus*, and *Rhipicephalus sanguineus* [102]. Many species of ticks belonging to the genus *Ixodes* are involved in Lyme disease, considered the most prevalent VBD in the United States and Europe [103], which is usually transmitted by *Ixodes* *scapularis* and *Ixodes pacificus* in North America and Central America and by *Ixodes persulcatus* and *I. ricinus* in Europe and Asia [104]. The articles we evaluated indicated that the global population of *I. ricinus* in Europe could duplicate by 2080, even expanding to areas to the north and east of their current distribution range, reaching more septentrional regions, such as Sweden and Russia [105]. Boeckmann and Joyner [106] conducted an analysis based on the model of distribution records and defined future predictions that included Europe, the Iberian Peninsula, and Scandinavia. In the United States, the models suggest a higher probability of the emergence of *I. scapularis* in the Gulf of Mexico region. Future projections point toward stabilization of the population’s range of distribution by 2050 [107].

Concerning the ecological conditions necessary for tick growth, many studies have proposed that microclimates play a role in their abundance. Such studies consider climate conditions at a micro-scale, measured in specific areas, for example, just above the soil surface (including temperature, wind speed, level of exposition, saturation deficit, and soil humidity) [108]. Microclimate variations may increase or decrease tick survival and development rates and induce changes in their population growth, behavior, susceptibility to pathogenic agents, pathogen incubation period, seasonal variations in biting habits, and pathogen transmission capacity [12, 108].

### Variables and risk factors associated with VBDs

In Table-3, climate variables related to VBD transmission are summarized based on data extracted from the evaluated articles and classified into six subgroups: Air temperature, precipitation, humidity, extreme climate events, climate variability, and others.

**Table-3:** Classification of climatic variables associated to vector-borne diseases.

Subgroup	Climate type
Air temperature	
General	Temperature
	Temperature above the average range
	Average temperature
	Highest temperature
	Lowest temperature
	Temperature range
	Cooling degree temperature
Changes	Temperature changes
	Temperature increase or decrease
	Median increase of temperature
	Increase in the highest temperature
	Increase in the lowest temperature
	Increase or decrease in the temperature range
	Increase or decrease in spring/summer/autumn/winter temperatures
	Global warming
Seasonality	Summer or winter temperatures
	Temperatures in February or May
Precipitation	
General pattern	Precipitation
	Average precipitation
Changes	Changes in precipitation
	Increase or decrease in precipitation
	Precipitation anomalies
Seasonality	Precipitation in Apr/May/Jul/Oct/Nov
	Precipitation seasonality
	Dry/Hot/Humid season precipitation
	Summer or autumn precipitation
Humidity	Absolute humidity
	Relative humidity
Extreme climatic events	Drought
	Dust
	Flood
	Heat
	Extended autumn
	Extreme precipitation
Climatic variability	El Niño
	El Niño – Southern Oscillation
	North Atlantic Oscillation
	Pacific Decadal Oscillation
	Southern Oscillation Index
	Climatic seasonality
	Climatic variability
Others	Air pressure
	Soil-water content
	Hours of daylight
	Ultraviolet radiation
	Wind speed

Although climate change is a well-established global phenomenon, its effects vary according to geography [109]. So far, research has mainly been concentrated in the developed world, such as European countries and the United States, or those countries that have increased their research investment, like China. In addition, regions with increasing temperatures have received more attention from investigators, and few studies have been conducted in regions with more significant temperature variability.

Although VBDs have been more commonly studied in tropical zones, a higher climate impact is not necessarily driven by temporal changes in temperature variability over many decades as a result of short-term variations caused by extreme climate changes [110]. We have observed that many studies show that short-term temperature variations may affect the emergence, dissemination, persistence, and resurgence of VBD outbreaks and directly affect the survival rates of pathogenic agents outside their host as well as their dissemination [111]. Climate variations driven by El Niño-Southern Oscillation have been correlated with increases in VBD transmission cycles, as in the cases of dengue fever [112], malaria [113], hemorrhagic fever with renal syndrome [114], leishmaniasis [115], Lyme disease [116], and rickettsiosis [117], among others. Climate variations may also indirectly affect disease transmission probabilities as they change the distribution behavior of host or vector populations [118].

According to the Intergovernmental Panel on Climate Change, floods and their adverse effects, regarded as extreme events, have not been studied adequately nor frequently enough in the most vulnerable regions of the world as disasters connected to climate change and infectious disease outbreaks [110]. Historical records exist documenting infectious disease outbreaks that have emerged after extreme meteorological phenomena, particularly floods [119]. In Pakistan, nationwide floods in 2010 resulted in millions of medical consultations for gastroenteritis, respiratory tract infections, and malaria 2 months after the event occurred [120, 121]. Likewise, West Nile fever cases resurged in Europe and United States after a flood event [122].

Changes in precipitation patterns have been associated with diseases transmitted by mosquitoes and ticks, such as dengue, West Nile fever, Japanese encephalitis, Chikungunya virus, malaria, Lyme disease, and hantavirus infections, mainly when such changes coincide with high temperatures [123–125]. Fouque and Reeder[126] stated that fluctuations in precipitation patterns could influence the dynamics of mosquito populations and the transmission of mosquito-borne diseases. Thus, a variation in rainfall patterns from moderate to heavy may synchronize with the activity of mosquito populations, increasing humidity levels near the surface and stimulating gravid females at rest to oviposit and subsequently look for new hosts [126]. Qiao *et al*. [76] conducted a field study from which they inferred that a precipitation level >350 mm/d, as well as temperature and humidity conditions, probably contribute to the formation of the vector’s habitat. Thus, it could be possible to set an upper threshold value for a positive effect of precipitation on disease vectors [119]. Risk factors for the emergence of diseases after floods include the contamination of potable water artificial reservoirs, the expansion in the number and range of vector habitats, and changes in human activity [127].

Analysis of the scientific literature in this work showed significant associations between environmental and climate variables and a higher incidence of infectious diseases transmitted by vectors in various regions (Table-4) [89, 107, 128–131].

**Table-4:** Factors associated with vector-borne diseases.

Risk factors, by theme	OR (CI 95%)	Geographic region	Reference
Variables associated with climatic variability			
High temperatures	1.73 (1.02–2.92)	United States Singapore	[107]
Extreme temperatures and precipitation	1.4 (1.1–1.8)	Nepal Argentina	[128]
Rain season	2.2 (1.6–3.1)		
High relative humidity (>80%)	1.4 (1.1–1.8)	Colombia	[129]
Highest temperature	1.4 (1.0–2.0)	Cameroon, Africa Mexico	[130]
Lowest temperature	1.6 (1.0–2.4)		
Drought	2.6 (1.5–6.45)	Nepal	[131]
Vector-related variables			
Presence of the vector	1.025 (1.015–1.035)	São Paulo, Brazil Spain	[89]
Presence of ticks in hosts	1.939 (0.999–3.764)		
Common areas of biting insects	10.3 (2.3–45.5)	Cameroon, Africa	[130]
Variables related to geography			
High-elevation landform	1.4 (1.2–1.8)	Nepal	[128, 131]
Urban/Peri-urban location	1.515 (1.036–2.231)	São Paulo, Brazil	[89]
Presence of crops, wild and domestic animals	1.759 (1.028–3.003)		
Land use (crops)	1.04 (1.008–1.068)	Cameroon, Africa	[130]

CI=Confidence interval

The complexity of the interactions between climate, biotic/abiotic variables, and non-climate factors vary considerably depending on the type of disease and the particular location. VBDs are among the most studied illnesses in relation to climate and environmental aspects due to their high disease burden, their extended presence in tropical and subtropical areas, and their high sensitivity to variations in the climate and environment [12, 119, 125], in contrast with other risks, such as high-temperature stress, or exposure to storms and floods [116]. The closest association observed involves temperature, which affects vector biting and stinging rates, their survival and growth, and the survival and development of the pathogens they bear [132]. Precipitation also significantly impacts diseases transmitted by vectors that undergo aquatic stages in their life cycle (e.g., mosquitoes). Finally, humidity levels have also been shown to influence diseases transmitted by vectors such as ticks and sandflies [123, 133].

Climate conditions exert a wide range of indirect effects on the environment and human systems that favor the transmission of VBDs. Droughts may influence water storage, land use, irrigation practices, population movements, and vector ecology, driving changes in vector geographical distribution and human exposure to infection [116, 134]. According to the World Health Organization, the geographical distribution of VBDs has remained relatively stable; however, this has currently been changing due to different factors, such as global warming, intensive farming, dam building, irrigation, deforestation, population movements, non-planned urbanization, and the dramatic increase in travel and international commerce (globalization) [135].

In support of the closeness and scope of these connections, it is possible to find observational evidence of meteorological factors as well as seasonal and interannual patterns of incidence of diseases in specific locations. Thus, climate variables can be proposed as a solid argument to explain the geographic distribution of most, if not all, VBDs.

## Conclusion

Improving our understanding of the relationship between VBDs and related environmental and ecological conditions could provide an essential first step toward finding solutions to mitigate the vulnerability produced by these diseases and their general impact on communities.

Global warming, caused by both natural and anthropogenic factors, interacts in the long-term with natural variability and influences the transmission patterns of VBDs, in the short-term (years) and also long-term (decades), producing variable effects at different times and different places.

Based on our systematic review, the research predicts that the more global temperatures increase, so does the potential trend toward the occurrence of extreme events; therefore, determining the role of climate and environmental variations on the incidence of infectious diseases is a public health priority.

Only few studies [12, 119, 125] have explored the relationship between ecological (biotic and abiotic) and environmental (water, atmosphere, fauna, flora, and landscape) factors and VBDs. We found that most of the observations were based on case reports and some local databases, so analysis of the relationship was only focused on specific areas. VBDs with few data points were excluded from those analyses.

This systematic review allowed us to collect information on VBDs and their associations and identify knowledge gaps from a spatial and temporal perspective. Although this search was not exhaustive in compiling all the information on this topic, the articles analyzed can be considered representative of research trends. Our analysis of the information extended beyond individual cases to generate a series of relevant observations that could be applied in different models.

### Study limitations

It is difficult to generalize our knowledge of VBDs from a geospatial point of view, mainly because every case is inherently independent with regard to variable selection, geographic coverage, and temporal extension. The observational and retrospective nature of systematic reviews make them vulnerable to biases, and a systematic review cannot include as many studies as a narrative review.

## Authors’ Contributions

LPM, LAG, and LAR: Conception and design. LPM, LAG, and LAR: Data collection, data analysis, and interpretation. LPM, LAG, and LAR: Drafted the manuscript. All authors have read and approved the final manuscript.
